# Idiopathic Nephrotic Syndrome: Characteristics and Identification of Prognostic Factors

**DOI:** 10.3390/jcm7090265

**Published:** 2018-09-09

**Authors:** Charlotte Dumas De La Roque, Mathilde Prezelin-Reydit, Agathe Vermorel, Sébastien Lepreux, Colette Deminière, Christian Combe, Claire Rigothier

**Affiliations:** 1 Service de Néphrologie Transplantation et Dialyse, Centre Hospitalier Universitaire de Bordeaux, Hôpital Pellegrin, 33076 Bordeaux, France; mathilde.reydit@gmail.com (M.P-R.); agathe.vermorel@chu-bordeaux.fr (A.V.); christian.combe@chu-bordeaux.fr (C.C.); claire.rigothier@chu-bordeaux.fr (C.R.); 2 University of Bordeaux, INSERM, Bordeaux Population Health Research Center, UMR1219, 33000 Bordeaux, France; 3 INSERM, Clinical Investigation Center-Clinical Epidemiology-CIC-1401, 33000 Bordeaux, France; 4 Aurad-Aquitaine, Service Hémodialyse, 2 allée des Demoiselles, 33170 Gradignan, France; 5 Laboratoire de Pathologie, Centre Hospitalier Universitaire de Bordeaux, Hôpital Pellegrin, 33076 Bordeaux, France; sebastien.lepreux@ch-libourne.fr; 6 INSERM U1026, Université de Bordeaux, 33000 Bordeaux, France; 7 Laboratoire de Cytologie et d’Anatomie Pathologiques de Bordeaux, 30 bis rue Ulysse Gayon, 33000 Bordeaux, France; colette.deminiere@orange.fr

**Keywords:** idiopathic nephrotic syndrome, minimal change disease, focal segmental glomerulosclerosis, relapse, risk factors, complication

## Abstract

There are various histopathological forms of idiopathic nephrotic syndrome, including minimal change disease (MCD) and focal segmental glomerulosclerosis (FSGS). Whereas some relapse predictor factors have been identified in renal transplantation, the clinical future of idiopathic nephrotic syndrome in the native kidney remains uncertain. We designed a multicentric retrospective descriptive cohort study including all patients aged 15 years and over whose renal biopsy confirmed MCD or FSGS between January 2007 and December 2014. We studied 165 patients with idiopathic nephrotic syndrome; 97 with MCD and 68 with FSGS. In the MCD cohort, 91.7% of patients were treated with corticosteroids for a median total duration of 13 months. During 45 months of follow-up, 92.8% of patients achieved remission and 45.5% experienced relapse. In this cohort, 5% of patients experienced terminal kidney disease. With respect to FSGS patients, 51.5% were treated with corticosteroids for a median total duration of 15 months. During 66 months of follow-up, 73.5% of patients achieved remission and 20% experienced relapse. In this cohort, 26.5% of patients experienced terminal kidney disease. No statistical association was observed between clinical and biological initial presentation and relapse occurrence. This study describes the characteristics of a cohort of patients with the nephrotic idiopathic syndromes of MCD and FSGS from the time of renal biopsy and throughout follow-up.

## 1. Introduction

Idiopathic nephrotic syndrome (INS) accounts for 15–30% of adult glomerulopathies [1]. The anatomopathological presentations described involve minimal change disease (MCD), focal segmental glomerulosclerosis (FSGS), diffuse mesangial sclerosis, and membranous nephropathy. Whereas the pathophysiology of idiopathic nephrotic syndrome remains unclear, common features are based on podocyte alterations with protein expression or localization defects, actin cytoskeleton remodeling, or intracellular signaling pathway activation. Taken together, alterations lead to a disturbance of glomerular filtration barrier properties.

Besides protein mutations, the existence of circulating permeability factor(s) in the plasma of patients with idiopathic nephrotic syndrome was suggested a few years ago through different observations: resolution of FSGS after kidney transplantation from a donor with FSGS to a healthy recipient [2]; efficacy of plasmatic exchanges and/or immunoadsorption to obtain INS remission in native or transplanted kidney disease [3,4]; transient mother-to-fetus proteinuria transmission [5]; and murine models in which injection of T lymphocyte supernatant or serum from patients with INS induced proteinuria with histological MCD or FSGS lesions [6,7]. However, the hypothetical circulating permeability factor(s) were still not successfully identified. Various molecules were highlighted but none were found to have a sufficient level of evidence. The factor(s) could be a protein with a molecular weight of 30–50 kDa, with a strong affinity to galactose and protein A. In MCD or FSGS, VPF [6], IL-13 [8], and hemopexin [9], as well as suPAR [10], CLCF-1 [11], and CD40 antibodies [12] have all been mentioned. The occurrence of the disease in the context of immune challenge (infection, allergy) emphasized the possible link between immunity and INS [13]. INS is associated to a cytokine environment leading to abnormal T-cell response and abnormal cooperation between T- and B-cells [14].

Despite several advances in INS pathophysiology, MCD and FSGS still remain complex diseases for which new therapies are needed. Clinical evolution is still unpredictable. No cohort study has succeeded in bringing out prognostic factors. In adults, few retrospective cohort studies describe epidemiology, treatment responses, and relapses. Data for complications are scarce. Few studies have tried to identify prognostic factors of remission, relapse, or evolution of renal function. Nolasco et al. [15] and Mak et al. [16] found an association between age and frequency and time of relapse. Younger patients presented more frequently with earlier relapses. This was associated with a faster response to treatment and a premature withdrawal of steroids. Recently, Lee et al. [17] found a reduction in the risk of relapse with increasing age (OR 0.79; 95 CI 0.65–0.96; *p* = 0.017), a combination of corticosteroid and cyclophosphamide therapy (OR 0.36, CI 0.13–0.95; *p* = 0.039), and treatment duration (OR 0.91, CI 0.87–0.97; *p* = 0.001). In renal transplant patients, risk factors for idiopathic FSGS relapse have been clearly identified: age <15 years, rapid renal failure within the 3 years following diagnosis, and history of relapse in a previous transplantation.

To our knowledge, no large prospective study has been published to describe INS, MCD, or FSGS in adults according to the KDIGO guidelines published in 2012 [18].

In this study, we constituted and described a cohort of idiopathic nephrotic syndromes, MCD and FSGS, in adults. We aimed to identify association factors between initial clinical or biological presentation and relapse occurrence.

## 2. Experimental Section

### 2.1. Study Design and Population

We designed a multicentric retrospective cohort of adult INS with MCD or FSGS proven by histopathological analysis in nine nephrology centers in Nouvelle Aquitaine, France. All patients aged 15 years and over who had had a renal biopsy diagnosing MCD or FSGS between January 2007 and December 2014 were included. Renal graft biopsies and patients with less than two months follow-up were excluded. All biopsies from this region were analyzed by two pathologists (SL and CD) and secondly reviewed by an independent pathologist, which means that our cohort can be considered as exhaustive. Pathologists used currently admitted definitions of MCD and FSGS. 

### 2.2. Clinical Data

All medical files were studied to collect clinical and biological data from the time of renal biopsy to the end of the follow-up. Data were collected every month during the first six months and then every six months. Information was also compiled on thrombosis and familial renal history, as well as immunosuppressive and antihypertensive therapy.

### 2.3. Definitions

We used the French definitions according to the “*Haute Autorité de Santé*” National Protocol for Diagnosis and Care (PNDS) guidelines for adult idiopathic nephrotic syndrome. Complete remission was defined as protein excretion below 0.3 g/day or a protein–creatinine ratio of 0.3 g/g. Partial remission was defined as a protein excretion between 0.3 and 3 g/day (or 0.3–3 g/g). Relapse was defined as recurrence of proteinuria above 3 g/day (or 3 g/g). Steroid dependence was defined as a relapse in the 2 weeks after stopping treatment. Steroid resistance was defined as persistence of proteinuria above 3 g/day despite steroid therapy with 1 mg/kg/day for at least four months. Estimated glomerular filtration rate (eGFR) was calculated using the Modification of Diet in Renal Disease equation (MDRD). 

### 2.4. Statistical Analysis

Baseline characteristics were expressed as median and interquartile range (IQR). Descriptive statistics of the cohort for clinical and biological data, treatments, relapse, complications were performed using paired *t*-test or Mann–Whitney test as appropriate, with Prism GraphPad software. To identify risk factors for relapse in MCD, a univariate logistic regression was performed to study the impact of several parameters of initial presentation (clinical and biological data) on relapse occurrence. A multivariate Cox model was assessed with Statistica software to study the impact of the initial feature on relapse-free survival.

## 3. Results

### 3.1. Clinical Characteristics

**Figure 1:**
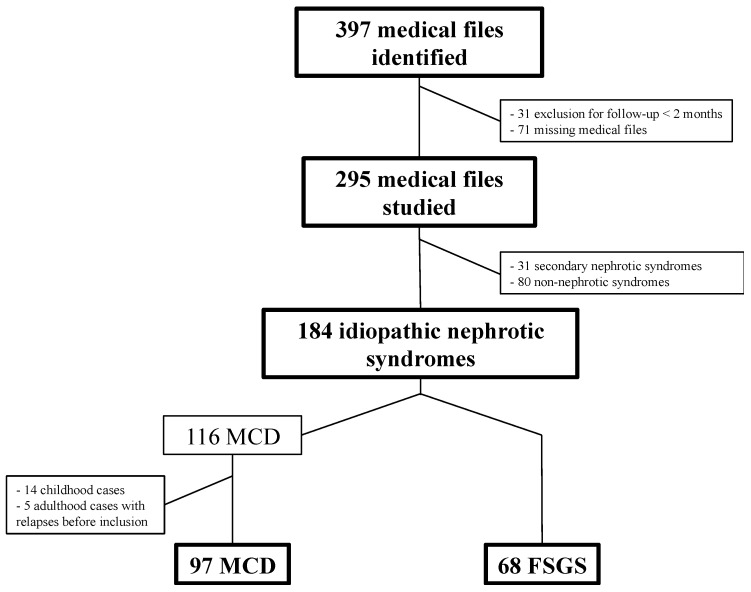
Flow chart. MCD: minimal change disease; FSGS: focal segmental glomerulosclerosis.

From 397 biopsies with a diagnosis of MCD or FSGS, we included 295 patients with 184 idiopathic forms, 31 secondary forms, and 80 non-nephrotic syndromes. We secondarily excluded idiopathic nephrotic syndromes that had begun during childhood (*n* = 14) and cases of adult INS with two or more relapses before the inclusion biopsy (*n* = 5). Figure 1 shows the flow chart of this study.

For idiopathic MCD, the median age was 47 years. Hypertension was present in 48.8% of patients. Initial median eGFR, albumin, and proteinuria values were respectively 79 mL/min/1.73 m^2^ (IQR 48–103), 21 g/L (IQR 16–23.5), and 6.5 g/day (IQR 4.2–11). Besides the 97cases of idiopathic MCD, we observed 17 secondary forms, with median age 52 years and 7.1% with hypertension. Median eGFR, albumin, and proteinuria values were respectively 79 mL/min/1.73 m^2^ (IQR 46.5–96), 28 g/L (IQR 19.5–36.7), and 5.8 g/day (IQR 3.6–8.2).

The idiopathic FSGS population (68 patients) presented a median age of 57 years. Hypertension was present in 46.3% of patients. Initial median eGFR, albumin, and proteinuria values were respectively 42.5 mL/min/1.73 m^2^ (IQR 29–58), 29 g/L (IQR 23–37), and 6 g/day (IQR 3.9–9). In addition, 14 secondary forms were observed, with median age of 51.5 years, and 41.7% of participants had hypertension. Median eGFR, albumin, and proteinuria values were respectively 50 mL/min/1.73 m^2^ (IQR 30–80), 32 g/L (IQR 21.5–35.7), and 6.8 g/day (IQR 3.8–11.3).

Initial clinical and biological features of idiopathic nephrotic syndromes including MCD and FSGS are shown in Table 1.

### 3.2. Immunosuppressive Therapy

Overall, 91.7% of patients with MCD versus 51.5% of patients with FSGS received oral steroid therapy for median durations of 13 months (IQR 7–20.5) and 15 months (IQR 8–36), respectively. Methylprednisolone pulses were administered in 13.5% of MCD and 22.8% of FSGS patients. In addition, 21.3% and 17.6% of patients suffering from MCD or FSGS, respectively, were initially treated using an immunosuppressive combination based on corticosteroids. 

Alternative MCD treatments were used as first-line treatments: cyclosporine (30.9%) for a median duration of 25 months (IQR 10–43), mycophenolate mofetil (MMF) (18.6%) for a median duration of 19 months (IQR 5.7–30.5), and rituximab (9.3%) at a dose of 375 mg/m^2^, with a median of two injections administered (IQR 2–3). In addition, 33.3% of relapses were observed following rituximab administration. 

In FSGS, other therapy regimens were similar, with a variation in administration proportions: cyclosporine (3.8%) for a median duration of 13 months (IQR 5–26), mycophenolate mofetil (10.3%) for a median duration of 25 months (IQR 1.5–54), and rituximab (4.4%) at a dose of 375 mg/m^2^, with a median of four injections administered (IQR 2–4). Other immunosuppressive therapies were stopped in two-thirds of cases without relapse.

The different treatment regimens for idiopathic MCD and FSGS are summarized in Table 2.

### 3.3. INS Evolution

The median follow-up times for MCD and FSGS were 45 months (IQR 27–80.5) and 66 months (IQR 30–92), respectively. Remission was obtained in 92.8% of patients, with a median remission delay of 2 months. At 8 weeks, 74.7% achieved remission in the MCD population. Remission was obtained in 73.5% of patients (median delay of remission 3 months). At 12 weeks, 48.9% achieved remission in the FSGS population.

Additionally, 64.5% of spontaneous remissions were observed in the FSGS population, whereas two patients in the MCD population presented with spontaneous remission. 

Evolution of INS in MCD and FSGS is described in Table 3.

**Figure 2:**
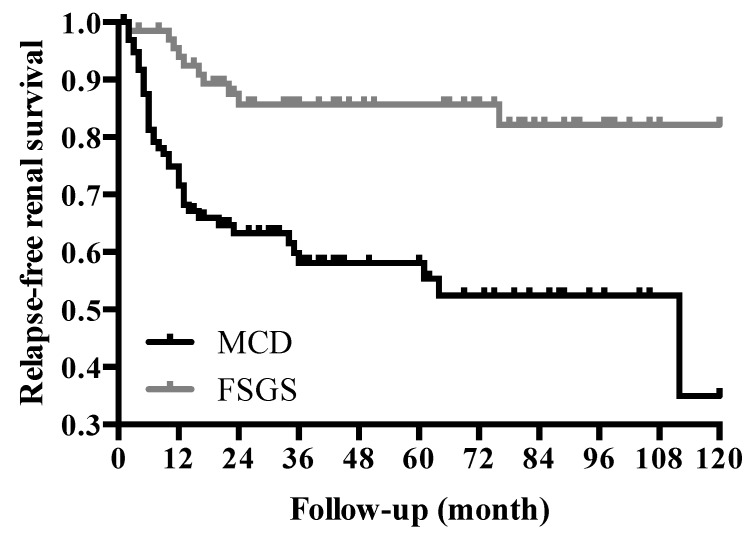
Relapse-free renal survival for MCD and FSGS patients. FSGS, focal segmental glomerulosclerosis; MCD, minimal change disease.

Relapse-free renal survival differed between the MCD and FSGS populations, with 40% and 15% of experiencing relapse, respectively (Figure 2).

### 3.4. Risk Factors

In patients with MCD, no statistical association was observed between initial presentation, age, eGFR, proteinuria, 8-week response, renin-angiotensin system (RAS) blockers use (angiotensin converting enzyme inhibitors (ACEi) or angiotensin receptor blockers (ARB)), and relapse occurrence. We did not find any association between initial presentation and relapse-free survival, even after adjustment for age, presence of hypertension, eGFR, albuminemia, presence of RAS blockade, and corticosteroid response at 8 weeks (Table 4).

Similarly, in the FSGS population, no statistical association between initial presentation, age, eGFR, proteinuria, 12-week response, RAS blockers, and relapse occurrence was observed. 

A multivariate Cox model was not performed due to the low number of events in this group.

### 3.5. Adverse Events

At the end of the follow-up, 3 patients were on hemodialysis, and 2 had an eGFR <15 mL/min/1.73 m^2^ in the MCD population, whereas 8 patients were on hemodialysis, 6 had received a renal graft, and 4 had an eGFR <15 mL/min/1.73 m^2^ in the FSGS population. Proteinuria and albuminemia evolved towards normalization. 

Overall, 4.3% of patients with MCD died during the follow-up, versus 5.9% in the FSGS population. We observed infectious events in 23.3% and 13.2%, steroid-induced diabetes mellitus in 11.2% and 11.4%, thrombosis in 7.2% and 2.9%, and neoplasia in 7.7% and 0% of the MCD and FSGS patients, respectively.

Adverse events for MCD and FSGS according to age are shown in Table 5. 

We observed that age is not significantly associated to drug-induced complications, except for infection, which occurs more often in middle-aged patients, probably because they are the group most often exposed to immunosuppressive regimens.

No statistical association was observed between duration of steroid therapy and occurrence of complications (infection, neoplasia, diabetes, thrombosis, death).

## 4. Discussion

We reported a complete description of a multicentric idiopathic MCD and FSGS population. The different cohorts published in the literature underlined the heterogeneity of medical practices. This may be partly due to the long study recruitment period with developments in treatment and recommendations. Most of the INS diagnoses were made before the KDIGO guidelines were published in 2012 [18]. We also observed incomplete biological criteria. Some FSGS cohorts define idiopathic nephrotic syndrome with albuminemia above 30 g/L, thus resulting in recruitment bias. Due to the design of our study based on histopathological findings, bias in recruitment and selection was drastically reduced. All the biopsies were analyzed twice by independent pathologists. Diagnoses were confirmed for 96% of patients. Some FSGS biopsies did not meet all FSGS criteria for instance number of glomeruli. Histopathological correlation was absent from this study, however, a histopathological study with biomarker analysis is in progress.

The follow-up times of 45 months for MCD and 66 months for FSGS enabled us to collect several data on short- and medium-term events. 

Our cohort was older than in previous published data, with a median age of 47 years old versus 27 to 50 for MCD, and 57 years old versus 30 to 47 for FSGS [19,20,21,22]. There was a large prevalence of hypertension, explained by the general epidemiology of hypertension. We observed stage 5 chronic kidney disease to be present in 5% of the MCD cohort, consistent with the reported prevalence of end-stage renal disease of 0–11% in the literature [15,16,17,18,19,20,21,22,23]. This rate was significantly higher for FSGS, at 26.5%. It has been proven that renal survival in FSGS is better in cases of remission than in cases of persistent nephrotic proteinuria [15,16,17,18,19,20,21,22,23,24,25,26,27,28]. The eight-week remission rate (74.7%) was comparable in our MCD cohort to the rates observed by Mak et al. and Waldman et al. (75.7% and 74.8%, respectively) [16,17,18,19,20,21,22,23,24,25]. Remission rates vary more widely in the literature, ranging from 30% to 82% [26,27]. The relapse rate in our study (45.5%) was comparable to previously reported rates: 48.5% for Fujimoto et al. and 44% for Huang et al., with similar follow-up times [19,28]. No statistical association between clinical and biological initial presentation and relapse occurrence was observed. Nolasco et al. [15], Mak et al. [16], and Lee et al. [17] found an association between age, corticosteroid use, duration of treatment, and the occurrence of relapse. The lack of reproducibility of previous results suggests insufficient statistical power and a weak association between these variables. Thus, a large prospective multicentric study is necessary to evaluate several variables with biological or histopathological parameters associated with relapse occurrence. 

In our population, 11% of subjects were observed to have steroid-induced diabetes mellitus. Few data on this complication have been reported in the literature, with a reported frequency ranging between 1% and 43% [23,25]. In the general population, steroid-induced diabetes mellitus is more frequent, with a proportion varying between 34% and 56% [29]. 

The incidence of neoplasia is low. To our knowledge, no study has analyzed the incidence or development of neoplasia in this specific cohort of INS. However, patients are exposed to long periods of immunosuppressive treatment. Longer follow-up is needed to decipher this complication.

Finally, the incidence of thrombosis confirmed previous observations with no supplemental risk factors identified. Nowadays, recommendations are based on the publication by Sarasin et al. in 1978. A randomized controlled therapeutic trial is needed to enable us to obtain evidence-based medical guidelines. The use of anticoagulation must be carefully evaluated, taking into account bleeding risk. Aspirin could be discussed here, based on the model of childhood INS disease or membranous nephropathy.

## 5. Conclusions

We report a French multicentric cohort of idiopathic minimal change disease and focal segmental glomerulosclerosis. This study described the clinical and biological characteristics of the nephrotic idiopathic syndromes MCD and FSGS from the time of renal biopsy and throughout follow-up. No predictive factor for relapse was identified by multivariate analysis. A multicentric prospective trial could be designed in future to identify strong prognostic factors.

## Figures and Tables

**Table 1:** Baseline clinical characteristics of patients with idiopathic nephrotic syndrome.

		Idiopathic MCD	Idiopathic FSGS	*p*
*n*	Total	97	68	-
	<30 years	26.80%	13.20%	
	30–59 years	39.20%	48.50%	
	≥60 years	34%	38.20%	
Age	Median (IQR)	47 (27.5–64)	57 (42.2–66.7)	0.01
Sex ratio	Men/women	55/42	48/20	-
Hypertension	Total	48.80%	46.30%	0.8
	<30 years	21.70%	33.30%	
	30–59 years	53.30%	25%	
	≥60 years	35.50%	80%	
Body mass index	Median	27	29	0.27
	(IQR)	(23–30)	(25–33)	
Genetic factor	Research Identification	7 (7.2%)	1 (1.5%)	-
		1 (1%)	0	
Familial INS		4 (4.1%)	0	-
Thrombosis history	Venous	3 (3.1%)	1 (1.5%)	-
	Arterial	3 (3.1%)	5 (7.3%)	
Cardiovascular risk factors	Median	1	1	-
	(IQR)	(0–1.5)	(1–2)	
Initial eGFR (MDRD, mL/min/1.73 m^2^)	Median (IQR)	79 (49–103)	42.5 (29–58)	<0.0001
	≥90	36.80%	6.20%	
	60–89	28.70%	12.50%	
	30–59	26.50%	51.60%	
	15–29	3.40%	28.10%	
	<15	4.60%	1.60%	
Proteinuria (g/day)	Median	6.5	6	0.4
	(IQR)	(4.2–11)	(3.9–9)	
Albuminemia (g/L)	Median (IQR)	21 (16–23.5)	29 (23–37)	<0.0001
	<30 g/L	88.20%	51.20%	
	<20 g/L	42.30%	13.90%	
Cholesterol (g/L)	Median	3.7	2.6	-
	(IQR)	(2.4–5)	(1.8–3.4)	
Triglycerides (g/L)	Median	1.9	2	-
	(IQR)	(1.2–2.7)	(1–3)	

FSGS, focal segmental glomerulosclerosis; MCD, minimal change disease; IQR, interquartile range; eGFR, estimated glomerular filtration rate; MDRD, modification of diet in renal disease; INS, idiopathic nephrotic syndrome.

**Table 2:** Therapeutic strategies of MCD and FSGS patients.

		Idiopathic MCD	Idiopathic FSGS	*p*
*n*		97	68	
Steroid therapy duration (months)	*n*	89 (91.7%)	35 (51.5%)	<0.0001
	First line	8 (5–14.7)	12 (8–18)	-
	Total	13 (7–20.5)	15 (8–36)	0.26
Other immune suppressors	Cyclosporine	37.10%	23 (33.8%)	0.7
	MMF	19.80%	7 (10.3%)	
	Cyclophosphamide	5.20%	1 (1.5%)	
	Tacrolimus	3.40%	0	
	Azathioprine	0.90%	0	
Rituximab	*n*	9 (9.3%)	3 (4.4%)	-
	Injections	2 (2–3)	4 (2–4)	
Anticoagulation indication	*n*	45 (46.4%)	12 (17.6%)	0.0002
	Atrial Fibrillation	2	7	
	Thrombosis	6	0	
	Albumin <20	35	5	
	Unknown	2	0	
RAS blockade	None	24 (24.7%)	3 (4.4%)	0.003
	ACEi	37 (38.1%)	32 (47.1%)	
	ARB	24 (24.7%)	14 (20.6%)	
	ACEi + ARB	12 (12.4%)	19 (27.9%)	

FSGS, focal segmental glomerulosclerosis; MCD, minimal change disease; RAS, renin-angiotensin system; MMF, mycophenolate mofetil; ACEi, angiotensin converting enzyme inhibitors; ARB, angiotensin receptor blockers.

**Table 3:** Remission and relapse in patients suffering from MCD or FSGS.

		Idiopathic MCD	Idiopathic FSGS	*p*
*n*		97	68	-
Follow-up	Median (IQR)	45 (27–80.5)	66 (30–92)	0.11
Remission		90 (92.8%)	50 (73.5%)	0.001
Remission (8 weeks)	Complete remission	43.40%	4.20%	-
	Partial remission	31.30%	44.70%	
	Total	74.70%	48.90%	
Relapse	*n*	41 (45.5%)	10 (20%)	0.003
Relapse number	Median (IQR)	2 (1–2.5)	1 (1–1.25)	-
First relapse delay (month)	Median (IQR)	7 (5.1–13.6)	14.5 (10.7–22.5)	0.06

FSGS, focal segmental glomerulosclerosis; MCD, minimal change disease; IQR, interquartile range.

**Table 4:** Multivariate analysis for relapse according to initial presentation in MCD.

Variable		Odds Ratio (OR)	Confidence Interval (CI) 95%	*p*
Age		0.97	0.94–1.01	0.14
Initial presentation	Isolated	1		
	Associated *	0.79	0.23–2.71	0.7
Hypertension		2.06	0.63–6.77	0.23
eGFR (MDRD)	<60	1		0.58
	60–90	0.52	0.14–1.89	
	>90	0.9	0.21–3.83	
Albuminemia	<20	1		0.91
	>20	0.94	0.32–2.76	
RAS blockade	None	1		0.3
	ACEi	2.16	0.5–9.39	
	ARB	3.41	0.63–18.70	
	ACEi + ARB	6.63	0.85–51.45	
Response (8 weeks)	Non responder	1		0.48
	Partial response	1.65	0.36–7.52	
	Complete response	2.47	0.56–10.81	

MCD, minimal change disease; RAS, renin-angiotensin system; ACEi, angiotensin converting enzyme inhibitors; ARB, angiotensin receptor blockers; eGFR, estimated glomerular filtration rate; MDRD, modification of diet in renal disease; * Associated with hypertension, renal failure, hematuria.

**Table 5:** Complications occurring in idiopathic MCD and FSGS.

Adverse Events	Histology	Total	<30 Years	30–59 Years	≥60 Years	*p*
Steroid-induced diabetes	MCD	11.20%	3.80%	11.10%	18.50%	0.24
	FSGS	11.40%	0%	5.50%	33.30%	0.07
	Total	11.30%	3.80%	8.90%	19.10%	0.16
Thrombosis	MCD	7.20%	3.80%	7.90%	9.10%	0.73
	FSGS	2.90%	0%	0%	7.70%	0.19
	Total		3.30%	5.60%	4.80%	0.95
Infection	MCD	26.80%	23.10%	31.60%	29.60%	0.03
	FSGS	13.20%	33.30%	3.30%	19.2	0.03
	Total		23.30%	19.40%	22.60%	0.86
Neoplasia	MCD	7.70%	3%	10.50%	9.10%	0.62
	FSGS	0%	-	-	-	-
	Total		0%	6.90%	4.80%	0.45
Death	MCD	4.30%	0%	2.60%	12.10%	0.07
	FSGS	5.90%	0%	3%	11.50%	0.28
	Total		0%	2.80%	11.10%	0.049

FSGS, focal segmental glomerulosclerosis; MCD, minimal change disease.

## References

[1] Audard V., Lang P., Sahali D. (2008). Pathogénie du syndrome néphrotique à lesions glomérulaires minimes. Médecine/Sciences.

[2] Rea R., Smith C., Sandhu K., Kwan J., Tomson C. (2001). Successful transplant of a kidney with focal segmental glomerulosclerosis. Nephrol. Dial. Transplant..

[3] Feld S.M., Figueroa P., Savin V., Nast C.C., Sharma R., Sharma M., Hirschberg R., Adler S.G. (1998). Plasmapheresis in the treatment of steroid-resistant focal segmental glomerulosclerosis in native kidneys. Am. J. Kidney Dis..

[4] Davenport R.D. (2001). Apheresis treatment of recurrent focal segmental glomerulosclerosis after kidney transplantation: Re-analysis of published case-reports and case-series. J. Clin. Aphere..

[5] Kemper M.J., Wolf G., Müller-Wiefel D.E. (2001). Transmission of glomerular permeability factor from a mother to her child. N. Engl. J. Med..

[6] Lagrue G., Xheneumont S., Branellec A., Hirbec G., Weil B. (1975). A vascular permeability factor elaborated from lymphocytes. I. Demonstration in patients with nephrotic syndrome. Biomedicine.

[7] Zimmerman S.W. (1984). Increased urinary protein excretion in the rat produced by serum from a patient with recurrent focal glomerular sclerosis after renal transplantation. Clin. Nephrol..

[8] Lai K.-W., Wei C.-L., Tan L.-K., Tan P.-H., Chiang G.S.C., Lee C.G.L., Jordan S.C., Yap H.K. (2007). Overexpression of interleukin-13 induces minimal-change-like nephropathy in rats. JASN.

[9] Lennon R., Singh A., Welsh G.I., Coward R.J., Satchell S., Ni L., Mathieson P.W., Bakker W.W., Saleem M.A. (2008). Hemopexin induces nephrin-dependent reorganization of the actin cytoskeleton in podocytes. JASN.

[10] Wei C., Trachtman H., Li J., Dong C., Friedman A.L., Gassman J.J., McMahan J.L., Radeva M., Heil K.M., Trautmann A. (2012). Circulating suPAR in two cohorts of primary FSGS. JASN.

[11] McCarthy E.T., Sharma M., Savin V.J. (2010). Circulating permeability factors in idiopathic nephrotic syndrome and focal segmental glomerulosclerosis. Clin. J. Am. Soc. Nephrol..

[12] Delville M., Sigdel T.K., Wei C., Li J., Hsieh S.-C., Fornoni A., Burke G.W., Bruneval P., Naesens M., Jackson A. (2014). A circulating antibody panel for pretransplant prediction of FSGS recurrence after kidney transplantation. Sci. Transl. Med..

[13] Shalhoub R.J. (1974). Pathogenesis of lipoid nephrosis: A disorder of T-cell function. Lancet.

[14] Grimbert P., Audard V., Remy P., Lang P., Sahali D. (2003). Recent approaches to the pathogenesis of minimal-change nephrotic syndrome. Nephrol. Dial. Transplant..

[15] Nolasco F., Cameron J.S., Heywood E.F., Hicks J., Ogg C., Williams D.G. (1986). Adult-onset minimal change nephrotic syndrome: A long-term follow-up. Kidney Int..

[16] Mak S.K., Short C.D., Mallick N.P. (1996). Long-term outcome of adult-onset minimal-change nephropathy. Nephrol. Dial. Transplant..

[17] Lee H., Yoo K.D., Oh Y.K., Kim D.K., Oh K.-H., Joo K.W., Kim Y.S., Ahn C., Han J.S., Lim C.S. (2016). Predictors of relapse in adult-onset nephrotic minimal change disease. Medicine.

[18] National Kidney Foundation (2012). KDIGO Clinical Practice Guideline for Glomerulonephritis. Kidney Int. Suppl..

[19] Fujimoto S., Yamamoto Y., Hisanaga S., Morita S., Eto T., Tanaka K. (1991). Minimal change nephrotic syndrome in adults: Response to corticosteroid therapy and frequency of relapse. Am. J. Kidney Dis..

[20] Fernandez-Juarez G., Villacorta J., Ruiz-Roso G., Panizo N., Martinez-Marín I., Marco H., Arrizabalaga P., Díaz M., Perez-Gómez V., Vaca M. (2016). Therapeutic variability in adult minimal change disease and focal segmental glomerulosclerosis. Clin. Kidney J..

[21] Stirling C.M., Mathieson P., Boulton-Jones J.M., Feehally J., Jayne D., Murray H.M., Adu D. (2005). Treatment and outcome of adult patients with primary focal segmental glomerulosclerosis in five UK renal units. QJM.

[22] Greenwood A.M., Gunnarsson R., Neuen B.L., Oliver K., Green S.J., Baer R.A. (2017). Clinical presentation, treatment and outcome of focal segmental glomerulosclerosis in Far North Queensland Australian adults. Nephrology.

[23] Shinzawa M., Yamamoto R., Nagasawa Y., Oseto S., Mori D., Tomida K., Hayashi T., Izumi M., Fukunaga M., Yamauchi A. (2014). Comparison of methylprednisolone plus prednisolone with prednisolone alone as initial treatment in adult-onset minimal change disease: A retrospective cohort study. Clin. J. Am. Soc. Nephrol..

[24] Chun M.J., Korbet S.M., Schwartz M.M., Lewis E.J. (2004). Focal segmental glomerulosclerosis in nephrotic adults: Presentation, prognosis, and response to therapy of the histologic variants. JASN.

[25] Waldman M., Crew R.J., Valeri A., Busch J., Stokes B., Markowitz G., D’Agati V., Appel G. (2007). Adult minimal-change disease: Clinical characteristics, treatment, and outcomes. Clin. J. Am. Soc. Nephrol..

[26] Szeto C.-C., Lai F.M.-M., Chow K.-M., Kwan B.C.-H., Kwong V.W.-K., Leung C.-B., Li P.K.-T. (2015). Long-term outcome of biopsy-proven minimal change nephropathy in Chinese adults. Am. J. Kidney Dis..

[27] Nair R.B., Date A., Kirubakaran M.G., Shastry J.C. (1987). Minimal-change nephrotic syndrome in adults treated with alternate-day steroids. Nephron.

[28] Huang J.J., Hsu S.C., Chen F.F., Sung J.M., Tseng C.C., Wang M.C. (2001). Adult-onset minimal change disease among Taiwanese: Clinical features, therapeutic response, and prognosis. Am. J. Nephrol..

[29] Gonzalez-Gonzalez J.G., Mireles-Zavala L.G., Rodriguez-Gutierrez R., Gomez-Almaguer D., Lavalle-Gonzalez F.J., Tamez-Perez H.E., Gonzalez-Saldivar G., Villarreal-Perez J.Z. (2013). Hyperglycemia related to high-dose glucocorticoid use in noncritically ill patients. Diabetol. Metab. Syndr..

